# Rosin Derivative IDOAMP Inhibits Prostate Cancer Growth via Activating RIPK1/RIPK3/MLKL Signaling Pathway

**DOI:** 10.1155/2022/9325973

**Published:** 2022-08-04

**Authors:** Hong Xu, Xingkai Zeng, Xuecheng Wei, Zhongfeng Xue, Naiwen Chen, Wei Zhu, Wenhua Xie, Yi He

**Affiliations:** ^1^Department of Urology, The Affiliated Hospital of Jiaxing University, Jiaxing, China; ^2^College of Pharmacy, Guangxi University of Chinese Medicine, Nanning, China

## Abstract

Rosin derivatives such as dehydroabietic acid and dehydroabietic amine belonging to diterpenoids have similar structure with androgen that inhibited the occurrence and development of prostate cancer. In this study, the effects and possible mechanism of the rosin derivative IDOAMP on prostate cancer were investigated. Our results showed that IDOAMP effectively inhibited cell viabilities of LNCaP, PC3, and DU145 prostate cells. After the treatment with IDOAMP, the levels of cleaved-PARP, LC3BII/I, and HMGB1 were increased, whereas the expression of GPX4 was decreased. Interestingly, cell viability was reversed by the supplements of necrostatin-1 and necrosulfonamide. Meanwhile, the IDOAMP downregulated the expression of human Aurora kinase A that was overexpressed in prostate cancer. In addition, co-IP results showed that IDOAMP inhibited the binding of Aurora kinase A to the receptor-interacting serine/threonine kinase 1 (RIPK1) and RIPK3. However, the binding of RIPK1 to FADD, RIPK3, or MLKL was significantly promoted. Further studies showed that the phosphorylation levels of RIPK1, RIPK, and MLKL were increased in a concentration-dependent manner. In in vivo model, IDOAMP reduced the tumor volumes and weights. In conclusion, IDOAMP directly inhibited Aurora kinase A and promoted the RIPK1/RIPK3/MLKL necrosome activation to inhibit the prostate cancer.

## 1. Introduction

Prostate cancer (PCa), a malignant tumor of the male genitourinary system, is the 2nd most prevalent cancer affecting males [1], the 4th most frequently diagnosed cancer, and the 5th leading cause of death worldwide, comprising 14.1% of total new cancer cases and 6.8% of the total cancer-associated deaths in 2020 among males [2, 3]. Among all therapies for PCa, endocrine therapy is the most effective option [4], and three most commonly used endocrine drugs including goserelin, leuprorelin, and triptorelin are usually combined with bicalutamide or flutamide for the clinical treatment. Although these three endocrine drugs have shown the powerful anticancer properties owing to the structural similarity to luteinizing hormone-releasing hormone (LHRH), the resistance and other side effects of endocrine therapy would be unavoidable but appear eventually [5]. Therefore, developing effective therapies for PCa with less adverse effects is urgent.

Terpenoids are an important class of natural drugs or drug precursors for treating or preventing cancers and other diseases [6]. A previous study showed that *Ganoderma* triterpenoids significantly suppressed cell proliferation of two human PCa cell lines, LNCaP and PC3 [7]. Similarly, diterpenoids have been reported to possess biological activities against tumors particularly for PCa [8–10]. Moreover, numerous studies have shown that rosin derivatives extracted from plants exhibit antitumor effects [11–15]. Rosin derivatives such as dehydroabietic acid and dehydroabietic amine make up a large group of diterpenes. Dehydroabietic acid and dehydroabietic amine, which are two kinds of rosin derivatives, are typical diterpenes compounds. The rosin derivatives hydroabietic acid and dehydroabietic amine show similarities to androgen in their chemical structure, suggesting they may be active against PCa cells. Thus, we synthesized the rosin derivative 4-((((7-isopropyl-1,4a-dimethyl-1,2,3,4,4a,9,10,10a-octahydrophenanthren-1-yl)methyl)amino)methyl)phenol whose structure is similar to androgen. It is termed as IDOAMP because of its chemical structure, and we investigate that this IDOAMP has the potential to suppress PCa.

Previous studies showed that Aurora kinase A usually overexpressed in PCa is a direct negative regulator of necrosome activation, with its kinase activity necessary for binding to RIPK1/3 necrosome during necroptosis [16, 17]. Regulation of necrosis occurs through the programmed necrosis or necroptosis, which is emerging as an important target for cancer treatment [18–20]. Previous research reported that mediators including the receptor-interacting serine/threonine kinase 1 or 3 (RIPK1 or RIPK3) and mixed lineage kinase domain-like (MLKL) played a key role in necroptosis [21]. Based on the above, we have a conjecture that whether the IDOAMP can induce necroptosis via activating RIPK1/RIPK3/MLKL signaling pathway in PC3 cells. In this study, we have carried out the experiments and verified our conjecture. We propose that the IDOAMP emerged as a potential drug for the therapy of PCa.

## 2. Materials and Methods

### 2.1. Experimental Drugs and Reagents

Compound IDOAMP (Patent number: 2018101855645) was provided by Prof. Chunxin Lv from the College of Biological, Chemical Sciences and Engineering, Jiaxing University (Zhejiang, China). Anti-cleaved-poly (ADP-ribose) polymerase (PARP) antibody (cat_no.5625), anti-high-mobility group protein B1 (HMGB1) antibody (cat_no.6893), anti-phos-RIPK1 (Ser166) antibody (cat_no.44590), anti-RIPK1 antibody (cat_no.3493), and anti-MLKL antibody (cat_no.14993) were obtained from Cell Signaling Technology (Danvers, MA, USA). Antilipidation of microtubule-associated protein 1 light chain 3 (LC3B) antibody (ab192890), anti-phos-RIPK3 (Ser227) antibody (ab209384), anti-RIPK3 antibody (ab226297), anti-glutathione peroxidase 4 (GPX4) antibody (ab125066), anti-phos-MLKL (Ser358) antibody (ab187091), anti-Aurora kinase A antibody (ab52973), anti-FADD antibody (ab108601), goat anti-rabbit IgG H&L (horseradish peroxidase) (ab205718), and goat anti-mouse IgG H&L (horseradish peroxidase) (ab205719) were obtained from Abcam (Cambridge, UK). Anti-MLKL antibody (sc-293201) and anti-FADD antibody (sc-271520) were procured from Santa Cruz Biotechnology (Dallas, TX, USA). The Aurora A/Aurora B kinase enzyme system was procured from Promega (Madison, WI, USA). Necrostatin-1, necrosulfonamide, Z-VAD-FMK, ferrostatin-1, and chloroquine were obtained from MedChemExpress (Monmouth Junction, NJ, USA). Fetal bovine serum and F-12K were purchased from Gibco Thermo Fisher Scientific (Grand Island, NY, USA). Penicillin-streptomycin solution was procured from Sigma-Aldrich (St Louis, MO, USA). The hematoxylin-eosin (HE) staining kit was obtained from Beyotime Biotechnology (Shanghai, China).

### 2.2. Cell Viability

Cell viability of log-phase cells was detected by using the CCK8 assay kit. Briefly, the cells were seeded in 96-well plates (5,000 cells/well) followed by 24 h incubation at 37°C in a 5% CO_2_-humidified environment. When the cells reached approximately 90% confluence, three cell lines (LNCAP, PC3, and DU145) were separately transferred to serum-free media and cultured for 2 h. The supernatants were then discarded, and the cells were treated using established protocols for another 24 h. Finally, the CCK-8 solution (10 *μ*L) was added to each well and incubated for 4 h, and the absorbance at 450 nm was measured using a fluorescent microplate reader (Bio-Rad, USA). Cell proliferative ability was determined as follows: cell growth inhibition rate = 1–(experimental group OD value/control group OD value) × 100%.

### 2.3. Western Blot

The cells were washed for three times in PBS and then lysed using the RIPA lysis buffer at 4°C. Protein samples were denatured by boiling for 10 min, and protein concentrations were assessed using BCA kits (Pierce BCA; Thermo Scientific). Proteins were subjected to 8% sodium dodecyl sulfate-polyacrylamide gel electrophoresis and transferred to polyvinylidene fluoride (PVDF) membranes. Membranes were blocked using 5% (*m*/*v*) fat-free milk dissolved in Tris-buffered saline with 5% (*v*/*v*) Tween-20 (TBS-T) for 1 h. Then, the membranes were incubated with primary antibodies diluted to the appropriate concentrations in TBS-T at 4°C overnight. After washing with TBS, secondary antibodies were diluted to 1 : 5000 in TBS-T and applied to the blots at room temperature for 1 h. Finally, the ECL reagent was used for blot imaging using a Tanon™ 6600 Luminescent Imaging Workstation (Tanon, China). Densitometric evaluation was conducted using the Image Pro Plus 6.0 software (Media Cybernetics Inc., Rockville, MD, USA). The *β*-actin was used as control, for determination of relative protein expression and phosphorylation levels.

### 2.4. Human Aurora Kinase A Activity Assay

Human Aurora kinase A activity assay was conducted at room temperature. In brief, 1 *μ*L of IDOAMP, 2 *μ*L of substrate/ATP mix, and 2 *μ*L enzyme were added into each well. The reaction proceeded for 60 min, until the ADP-Glo reagent (5 *μ*L) was then added to each well. Thereafter, the reaction proceeded for 40 min, followed by the addition of 10 *μ*L of kinase detection reagent. Finally, the reaction lasted for 30 min, and the luminescence signal was measured afterwards.

### 2.5. Co-Immunoprecipitation Assay

The cells were lysed with precooled RIPA lysis buffer. Total cellular proteins were extracted from PC3 cells and incubated with 5 *μ*L of primary antibodies (Aurora kinase A 1 : 50, RIPK1 1 : 100, IgG for the control group) at 4°C on a rocker overnight. Protein-A/G-agarose beads were added to capture antibody-antigen complexes. The antibody-antigen mixture was shaken slowly at 4°C overnight or for 1 h at RT. Immunocomplexes were obtained with protein-A/G-agarose beads and centrifugated at 14,000 × *g* for 5 s. Sediments were washed thrice in precooled PBS and resuspended in an appropriate volume of loading buffer, followed by 5 min of boiling. Detachments of eluted proteins were analyzed by western blot with appropriate antibodies.

### 2.6. Animal Procedures

Six-week-old BALB/c nude mice (*n* = 18, male, weight 18–20 g) were supplied by the Model Animal Research Center of Nanjing University (Nanjing, China). All mice were kept in a Laboratory Animal Center SPF grade animal room (23 ± 2°C, natural lighting, free diet). The mice were adaptively bred for 3 days and then xenografted with 3 × 10^6^ PC3 cells/mouse (subcutaneous injection). The mice were randomly divided into low-dose group (0.5 mmol/L IDOAMP), high-dose group (1 mmol/L IDOAMP), and control group (saline) after the tumor reached approximately 50 mm^3^. The mice were treated once a day for 20 consecutive days (intraperitoneal injection). Thereafter, the tumors, heart, liver, spleen, lungs, and kidneys were harvested, and tumor weight and volume were measured. Tumor dimensions were measured with calipers, and tumor volume was calculated as (*D* × *d*^2^)/2, where *D* and *d* represent the longest and shortest diameters, respectively.

### 2.7. HE Staining

Tumors were cut to 4 *μ*m thick sections and dried for 1 h in an oven at 60°C. The paraffin sections were dewaxed with xylene, and a gradient ethanol hydration was performed. Then, the sections were washed with distilled water, stained with hematoxylin, and rinsed under running water. The sections were then placed in 1% (*v*/*v*) hydrochloric acid ethanol until they appeared red and rinsed under tap water for blue color recovery. Subsequently, the sections were stained with eosin for 1 min and washed with tap water. Dehydration, hyalinization, and sealing were carried out with gradient ethanol, dimethylbenzene, and neutral gum, respectively. The sections were observed and photographed by inverted phase-contrast microscopy.

### 2.8. Statistical Analysis

Statistical evaluation was conducted using the GraphPad Prism 9.0 (GraphPad Company, USA). Groups of western blot data were compared by one-way ANOVA and Tukey's post hoc test. All data are expressed as means ± SD. *p* < 0.05 was the cut-off value for significance.

## 3. Results

### 3.1. IDOAMP Reduces the Viability of LNCaP, PC3, and DU145 Cells

**Figure 1:**
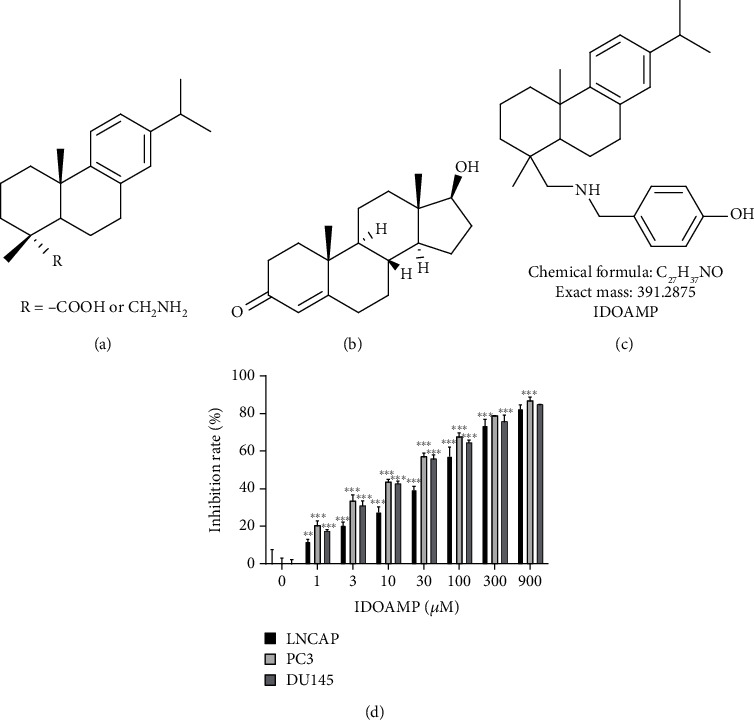
Structures of rosin derivative dehydroabietic acid and dehydroabietic amine (a), androgen (b), and IDOAMP (c). Growth inhibition of LNCaP, PC3, and DU145 cells in the presence of IDOAMP after 24 h treatment (d). (IDOAMP exhibited an inhibitory effect on LNCaP, PC3, and DU145 cells for drug groups at different concentrations compared with control group.) Data are presented as mean ± SD (*n* = 3). ^∗∗^*p* < 0.01 and ^∗∗∗^*p* < 0.001 vs. the control group.

Dehydroabietic acid and dehydroabietic amine are typical diterpenes compounds (Figure 1(a)). The rosin derivatives hydroabietic acid and dehydroabietic amine have similarities to androgen (Figure 1(b)) in their chemical structure, suggesting that they may be active against PCa cells. Therefore, we synthesized the IDOAMP (Figure 1(c)) whose structure is similar to androgen. Then, CCK-8 cell viability assays were conducted to assess the efficacy of IDOAMP against PCa cells. IDOAMP exhibited an inhibitory effect on LNCaP, PC3, and DU145 cells (Figure 1(d)). The IC_50_ values for IDOAMP against these cell lines were 53.34 ± 6.21, 16.53 ± 2.01, and 20.89 ± 0.82 *μ*M, respectively. These data indicate that the IDOAMP has an inhibitory effect on these three PCa cell lines.

### 3.2. RIPK1/MLKL May Be Involved in the Inhibitory Effect of IDOAMP on PC3 Cells

**Figure 2:**
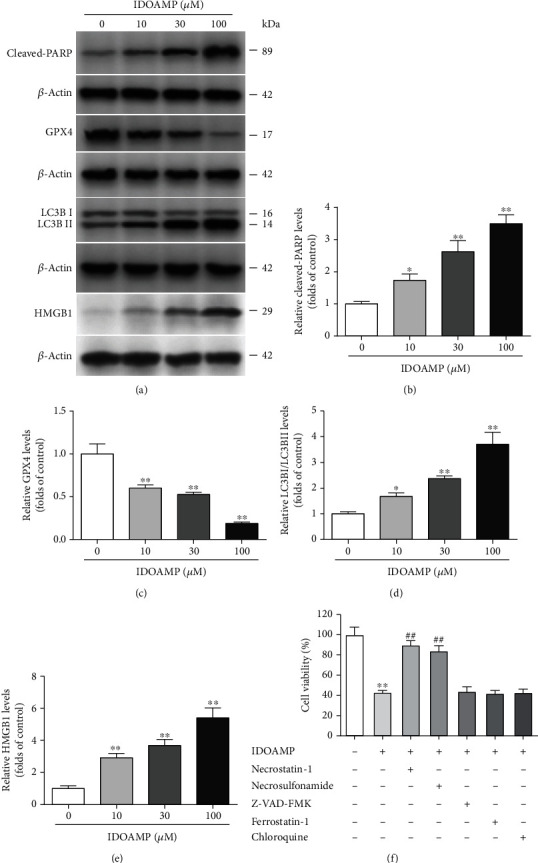
The protein expression level of cleaved-PARP, GPX4, LC3B, and HMGB1 in PC3 cells. (a) Representative bands. The protein expression levels of cleaved-PARP (b), GPX4 (c), LC3B (d), and HMGB1 (e) were normalized to those in the control-treated conditions. (f) PC3 cells were treated with IDOAMP in the absence or presence of the indicated inhibitors for 24 h, and then, cell viability was determined. The results are presented as mean ± SD (*n* = 3). ^##^*p* < 0.01, ^∗∗^*p* < 0.01, and ^∗^*p* < 0.05 vs. the control group.

Since IDOAMP showed a pronounced inhibitory effect on PCa, we then planned to study the involved mechanism of this inhibition on prostate cancers In this experiment, after treatment of PC3 cells with IDOAMP for 24 h, we tested the protein expression of biomarkers for apoptosis (cleaved-PARP), autophagy (LC3B-I/II), necroptosis/necrosis (HMBG1), and ferroptosis (GPX4) [17]. The results showed that the expression levels of cleaved-PARP, LC3B-II, and HMGB1 significantly increased, but the expression of GPX4 decreased (Figures 2(a)–2(e)). To explore which pathway is involved, we further used necrostatin-1 (inhibitor of RIPK1) [22], necrosulfonamide (an inhibitor of MLKL) [23], Z-VAD-FMK (caspase inhibitor) [24], ferrostatin-1 (inhibitor of ferroptosis) [25], and chloroquine (inhibitor of autophagy) [26]. Remarkably, cell viability in the necrostatin-1- and necrosulfonamide-treated groups was significantly restored, whereas Z-VAD-FMK, ferrostatin-1, and chloroquine had no significant effects on cell viability (Figure 2(f)), indicating RIPK1/MLKL may be involved.

### 3.3. IDOAMP Inhibits Aurora Kinase A and Promotes RIPK1/RIPK3/MLKL Necrosome Activation

**Figure 3:**
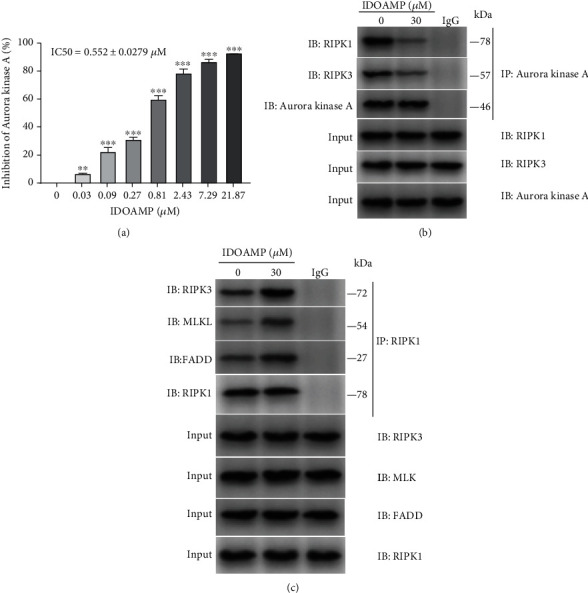
IDOAMP inhibits Aurora kinase A and promotes RIPK1/RIPK3/MLKL necrosome activation. (a) Inhibition of Aurora kinase A in the presence of IDOAMP after 24 h of incubation. (b) Co-IP analysis of the level of RIPK1 binding to FADD, RIPK3, and MLKL in PC3 cells. (c) Co-IP analysis of the levels of Aurora kinase A binding to RIPK1 and RIPK3 in PC3 cells following treatment with IDOAMP for 24 h. Data are presented as mean ± SD (*n* = 3). ^∗^*p* < 0.05, ^∗∗^*p* < 0.01, and ^∗∗∗^*p* < 0.001 vs. the control group.

Aurora kinase A is vital for centrosome maturation, meiotic maturation, spindle assembly, and metaphase I spindle orientation [27]. As described previously, Aurora kinase A was overexpressed in PCa [16]. The inhibitory effect of IDOAMP against human Aurora kinase A was determined using an ADP-Glo Kinase Assay Kit (tozasertib was elected as the positive control). Aurora kinase A was inhibited by IDOAMP (Figure 3(a)). These findings imply that human Aurora kinase A may be involved, and Aurora kinase A is crucial for necrosome activation, and its kinase activity is of importance in its binding to RIPK1/3 necrosome during necroptosis [17]. Thus, to investigate whether the IDOAMP affects the relationship between the Aurora kinase A and RIPK1 or RIPK3, we performed the co-IP assay. Our results showed that the IDOAMP inhibited the binding of Aurora kinase A to RIPK1 and RIPK3 in PC3 cells; meanwhile, the binding of RIPK1 to FADD, RIPK3 or MLKL, was significantly increased (Figures 3(b) and 3(c)). Collectively, IDOAMP may inhibit the binding of Aurora kinase A to RIPK1/RIPK3 but may promote RIPK1-RIPK3-MLKL necrosome formation.

### 3.4. IDOAMP Regulates RIPK1/RIPK3/MLKL Pathway

**Figure 4:**
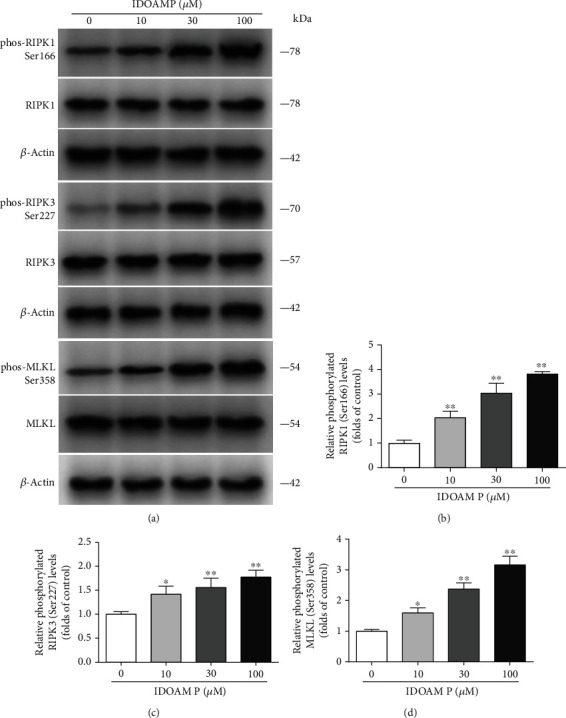
Effect of IDOAMP on RIPK1/RIPK3/MLKL signaling pathways in PC3 cells. (a) The phosphorylation levels of RIPK1, RIPK3, and MLKL in PC3 cells from different groups were detected by western blotting, and the representative bands are shown. Levels of RIPK1 (b), RIPK3 (c), and MLKL (d) were normalized to those in the control-treated conditions. The results are presented as mean ± SD (*n* = 3). ^∗^*p* < 0.05 and ^∗∗^*p* < 0.01 vs. the control group.

Necroptosis depends on the activation of a necrosome, a protein complex which is comprised of three core components: RIPK1, MLKL, and RIPK3 [28, 29]. After the supplementation with 10, 30, or 100 *μ*M of IDOAMP for 24 h, the phosphorylation levels of RIPK1, RIPK3, and MLKL in PC3 cells were significantly increased (Figure 4). This result suggests that IDOAMP may regulate the RIPK1/RIPK3/MLKL pathway.

### 3.5. IDOAMP Can Inhibit PC3 Xenograft Tumor Growth

**Figure 5:**
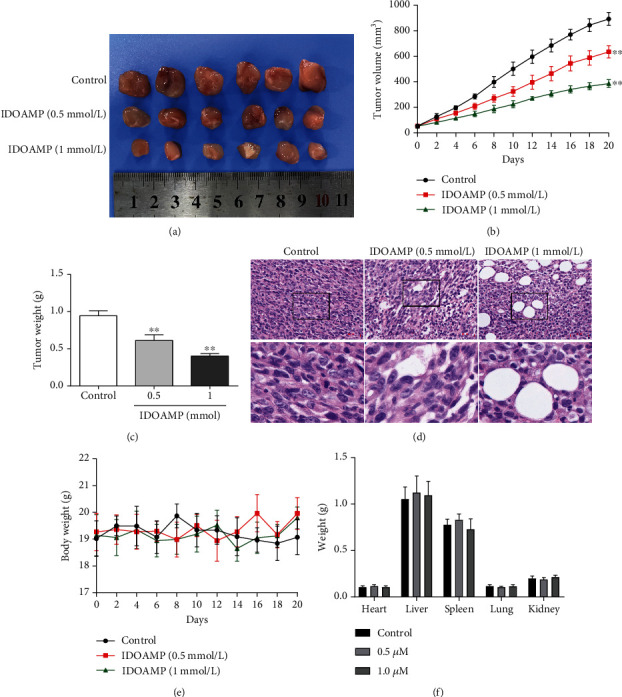
Tumor growth of PC3 xenograft-bearing nude mice. (a) Representative tumor morphology. (b) Tumor volume of the PC3 xenograft in the mice. (c) Effects on tumor weight. (d) HE staining of the tumor in nude mice (*n* = 6). (e) Graph shows the change in weight of the mice during the treatment period. (f) Organ weight of the mice. Weights of the heart, liver, spleen, lung, and kidney of the mice were shown as mean ± SD (*n* = 6). ^∗∗^*p* < 0.01 vs. the control group.

To investigate the effect of IDOAMP on tumor growth in vivo, IDOAMP was subcutaneously implanted into nude mice. Tumor volumes and tumor weights were significantly lower in the groups with IDOAMP than the control group (Figures 5(a)–5(c)). Moreover, the histopathological analysis revealed vacuolation in PC3 cells, representing steatosis, and the degree of vacuolation was correlated with IDOAMP (Figure 5(d)). These results demonstrated that the IDOAMP inhibits the tumor growth. In addition, there were no significant differences in body weight among the groups in 20 days (Figure 5(e)). The weight of heart, liver, spleen, lung, and kidney of mice from each group showed no significant differences (Figure 5(f)).

## 4. Discussion

**Figure 6:**
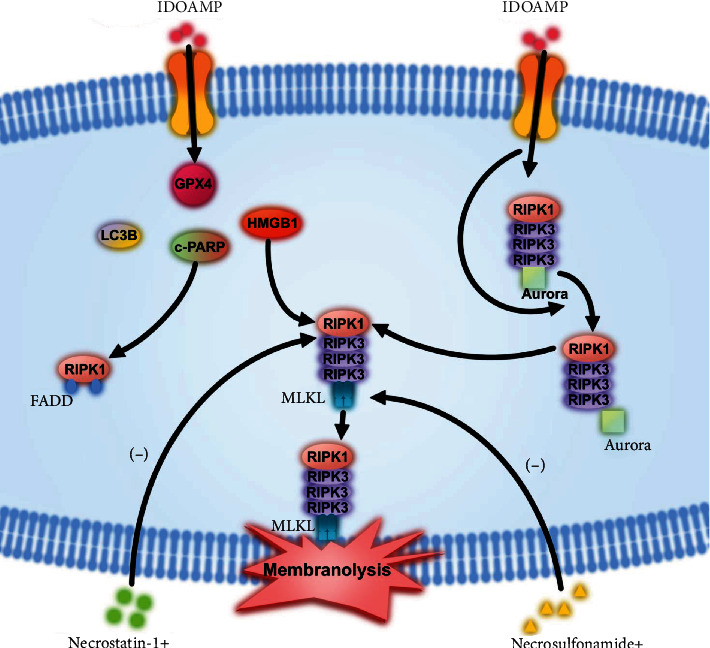
The mechanism of IDOAMP-induced inhibition of prostate cancer cell growth.

Several studies have shown that rosin derivatives have considerable multifaceted bioactivity, ranging from antibacterial and antianxiety effects to antiviral and anticancer actions [12, 13, 30]. In the present study, we found that a newly developed rosin derivative, which we termed as IDOAMP, exerted inhibitory effect on PC3 cell lines and inhibited the binding of Aurora kinase A to RIPK1 and RIPK3 as well as promoted RIPK1–RIPK3–MLKL necrosome formation to induce necroptosis. The mechanism of IDOAMP-induced inhibition of prostate cancer cell growth is summarized in Figure 6.

Recently, many approaches for inducing tumor cell death which are different from apoptosis, namely, autophagy, necroptosis, ferroptosis, and pyroptosis, have been developed. A previous study indicated that promoting apoptosis with androgen deprivation therapy (ADT) may be a cure for PCa [31]. However, ADT is associated with apoptosis resistance, leading to treatment-resistant PCa [32]. Moreover, upregulation of autophagy-related proteins promotes resistance to ADT [33, 34], suggesting that the autophagy machinery controls the transformations of cell death patterns between necroptosis and apoptosis [35]. Autophagic inhibition enhances the efficacy of abiraterone, a drug commonly used in PCa treatment [36]. In prostate cells with defective autophagy, toxic substances induce necroptosis rather than apoptosis [37]. Thus, suppressing autophagy and/or inducing necroptosis may increase the effectiveness of ADT [31]. Here, we demonstrated that IDOAMP induced PCa cell death with phenotypic features of autophagy, apoptosis, and necroptosis. As previously shown, the expression levels of cleaved-PARP, LC3B-II, and HMGB1 significantly increased, but only cell viability in the necrostatin-1- and necrosulfonamide-treated groups was significantly restored. This may result from the fact that Aurora kinase A is a direct negative regulator of necrosome activation that attaches importance to necroptosis. Meanwhile, our results showed that the IDOAMP inhibited the Aurora kinase A activity. Therefore, we concluded that the IDOAMP induces necroptosis rather than other forms of cell death in PCa. Nevertheless, necroptotic signaling played a vital role in mediating the anticancer activities of IDOAMP in PCa cells. Pharmacologic necroptosis inhibition considerably blocked IDOAMP-mediated PCa cell death.

Aurora kinase A is a constituent of the necrosome that can impede necroptosis by binding to RIPK1 and RIPK3 [17]. This is consistent with our results that IDOAMP showed obvious inhibitory effects against Aurora kinase A and reduced the binding of Aurora kinase A to RIPK1 and RIPK3. Thereafter, IDOAMP induced the necroptosis. At the same time, the IDOAMP increased the binding of RIPK1 to FADD, RIPK3, and MLKL. Thus, we speculate that IDOAMP inhibited Aurora kinase A and promoted necrosome activation of RIPK1/RIPK3/MLKL to induce necroptosis. However, necroptosis differs from apoptosis. The effect of necroptosis circumvents the development of multidrug resistance that results from the widespread use of chemotherapy drugs [17]. As apoptosis resistance may be a hallmark of cancer cells [38]. Our results showed that IDOAMP effectively activates necroptosis in PCa, and this may contribute to the avoidance of apoptosis resistance. Thus, IDOAMP has the potential for cancer treatments.

RIPK1/RIPK3/MLKL pathway-mediated necroptosis has been a research hotspot in recent years [39–41]. Necroptosis is modulated by RIPK1 that is a critical regulator [42]. When necroptosis is induced, RIPK1 can bind to RIPK3 via RHIM domains to phosphorylate RIPK1. Under the action of stimulating factors, RIPK3 is also phosphorylated. Next, p-RIPK3 forms a necrosome complex with p-RIPK1 to regulate necroptosis [40]. In our study, the results showed that IDOAMP directly inhibited the Aurora kinase A and reduced its binding to RIPK1 and RIPK3. A previous study has shown that the interaction between RIPK1 and RIPK3 is increased by the inhibition of Aurora kinase A, which is related to the enhanced RIPK-3-mediated phosphorylation of MLKL [17]. Phosphorylated MLKL is translocated to the plasma membrane and induces membrane rupture to precipitate the lethal step of necroptosis [43]. Our results showed that the IDOAMP induces necroptosis in PCa cells via activating RIPK1/RIPK3/MLKL signaling pathway, consistent with the findings of the above studies.

Our study showed that IDOAMP has anticancer effects both in vitro and in vivo. However, the specific mechanism of IDOAMP in vivo is in need of careful exploration. As we noted that these effects in humans have not been evaluated, further studies are necessary to determine the mechanism in vivo and potential for applying to clinic practice.

In summary, our results demonstrated that IDOAMP inhibited Aurora kinase A, promoting the RIPK1/RIPK3/MLKL necrosome activation to antiprostate cancer. Since IDOAMP has significant antitumor activities in vivo and in vitro, therefore, it possesses visible potential as drug candidate for prostate cancer treatment.

## Data Availability

The data used to support the finding of this study are included within the article.
